# Guillain–Barré/Miller Fisher overlap syndrome in a patient after coronavirus disease-2019 infection: a case report

**DOI:** 10.1186/s13256-021-03245-y

**Published:** 2022-02-08

**Authors:** Seyede Momeneh Mohammadi, Roghayeh Abdi, Zeinab Karimi, Fatemeh Mortazavi

**Affiliations:** 1 Department of Anatomical Sciences, School of Medicine, Zanjan University of Medical Sciences, Zanjan, Iran; 2 Imam Khomeini Hospital, Tabriz University of Medical Sciences, Tabriz, Iran; 3 Department of Anatomical Sciences, School of Medicine, Ardabil University of Medical Sciences, Ardabil, Iran

**Keywords:** GBS syndrome, COVID-19, Infection

## Abstract

**Background:**

Beyond the typical respiratory symptoms associated with novel coronavirus, increasing evidence has been reported of the neurological manifestations affecting both the central and peripheral nervous systems.

**Case presentation:**

We observed a 30-year-old Persian woman developing acute motor sensory axonal neuropathy, a variant of Guillain–Barré syndrome that overlaps Miller Fisher syndrome, 30 days after confirmed coronavirus disease-2019 infection. Our case highlight the rare occurrence of Guillain–Barré syndrome overlapping with Miller Fisher during the coronavirus disease-2019 pandemic. These neurologic manifestations may occur because of an aberrant immune response to coronavirus disease-2019.

**Conclusions:**

The early recognition of Guillain–Barré syndrome symptoms is critical, given the associated severe motor disabilities that may seriously limit the quality of life of these patients. We may still have much to learn about the co-occurrence of Guillain–Barré syndrome and Miller Fisher to improve the quality of life of these patients requiring an accurate evaluation by neurologists.

## Introduction

A new coronavirus, the severe acute respiratory distress syndrome–coronavirus-2 (SARS-CoV-2/ COVID-19), has spread fast throughout the world, leading to high morbidity and mortality [1]. COVID-19 is chiefly a respiratory infection, and the symptoms are related to the age and underlying medical condition of the patient and the immune system [2]. An increasing body of information reported neurological complications of COVID-19, including headache, dizziness, confusion, myalgia, and loss of taste and smell [3].

Mao *et al.* assessed neurological symptoms in 214 patients infected with COVID-19, and found that 36.4% of the patients exhibited neurological issues ranging from headache, dizziness, hyposmia, and muscle damage, to ischemic stroke [4]. Guillain–Barré syndrome (GBS) is an autoimmune disease of the peripheral nerves and nerve roots (polyradiculoneuropathy) that is usually caused by various infections such as *Campylobacter jejuni*, Epstein–Barr virus, influenza, and Zika virus [5, 6]. Miller Fisher syndrome (MFS) is a rare subtype of GBS and usually presents with at least two of the following features: ophthalmoplegia, areflexia, and ataxia. Some patients have weakness of the face, tongue, and swallowing muscles, as well as micturition disturbance. Others also develop weakness of the limbs and breathing muscles, and are then considered to have GBS-MFS overlap syndrome [7, 8].

GBS is characterized by ascending flaccid symmetrical limb paralysis with areflexia, sensory symptoms, and often involvement of the cranial nerves. Recently, some cases of GBS were reported in patients infected with COVID-19 [9–11]. We have little understanding of how COVID-19 infection results in GBS, and it needs to be investigated further. Although GBS syndrome is rare, the early diagnosis and treatment of GBS can considerably improve outcomes and avoid the need for ventilatory support. Here we report an acute motor sensory axonal neuropathy (AMSAN) case of GBS overlapped with MFS in a patient with COVID-19.

## Case presentation

### Patient information

A 30-year-old Persian woman presented weakness, stress, low blood pressure, and low-grade fever. On the tenth day after the onset of the symptoms, she complained of chest pain, cough, and tachycardia.

### Clinical findings

**Fig. 1:**
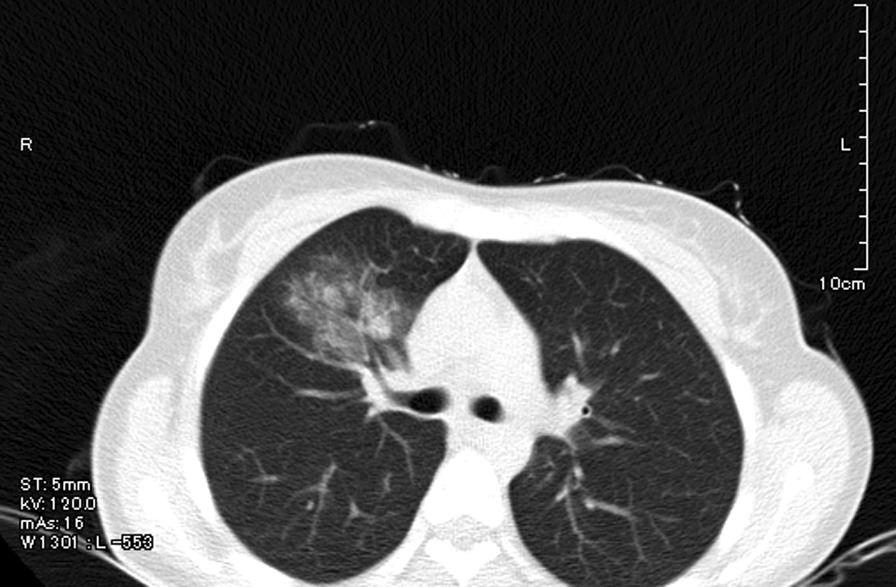
Chest computer tomography revealed the presence of unilateral ground-glass opacities

**Fig. 2:**
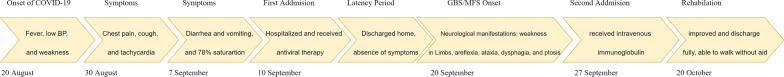
Timeline of clinical events, diagnostics/therapeutic approach, and clinical outcome

A chest computerized tomography (CT) scan revealed the presence of unilateral ground-glass opacities (Fig. 1). Following gastrointestinal complications, including diarrhea, vomiting, and 78% oxygen saturation, the patient was hospitalized and moved to the intensive care unit for invasive ventilation. She was treated with hydroxychloroquine, antiviral therapy (remdesivir), and tocilizumab. After partial recovery, the patient was discharged home. However, 30 days after the onset of symptoms, the patient manifested neurological complications (Fig. 2). She developed acute weakness in the lower limb, numbness and tingling, loss of touch and vibration sensation in the feet and, a few days later, in the upper limb and the hands. She also developed gait disorder and loss of balance. Subsequently, muscle stretch reflex examination revealed absent deep tendon reflexes in the upper and lower limbs. The patient also showed acute onset of unilateral eyelid ptosis (right), blurred vision (right), areflexia, dysphagia, vomiting, urinary incontinence (UI), and unilateral numbness of the chin and lower lip [numb chin syndrome (NCS)]. Electroneurography revealed severe sensory-motor axonal polyneuropathy with relative sparing of conduction velocities. The nerve conduction studies showed reduced or absent compound muscle action potentials (CMAP) and sensory nerve action potentials in the lower and upper limbs. Brain and spinal cord magnetic resonance imaging (MRI) did not reveal any abnormal and pathological findings. SARS-Cov-2 RNA was not tested in cerebrospinal fluid (CSF).

### Therapeutic intervention, follow-up, and outcome

The patient received intravenous immunoglobulin (IG) (0.4 g/kg/day for 5 days). The patient was highly responsive to treatment with the rapid clinical response on swallowing, strength, and eyelid ptosis. Also, the patient performed physical therapy successfully at her rehabilitation facility.

## Discussion

At the writing of this paper, SARS-CoV-2 has infected over 43 million individuals worldwide, with over 1.2 million deaths directly recognized to COVID-19 [12]. The virus belongs to the betacoronavirus family [13], and genetic analysis found its sequence is similar to SARS-CoV and MERS-CoV [14]. The most immediate causes of death involve acute respiratory distress syndrome (ARDS) and overwhelming shock due to cytokine storm [5]. As occurred in our patient, GBS is an acute areflexic paralytic disease that most commonly presents with progressive symmetric weakness. These symptoms generally develop 3 days to 6 weeks following an upper respiratory infection. The supposed pathophysiological mechanism is “molecular mimicry,” an aberrant autoimmune response to a primary infection that induces a cross-reaction against the peripheral nerve antigens. In this case, a clinical diagnosis of GBS was made based on the acute pattern of weakness, sensory dysfunction, and areflexia. In our case, the impairment of several cranial nerves in association with areflexia, ataxia, and demyelinating peripheral neuropathy suggests overlaps of MFS and GBS. Several variants of Guillain–Barré syndrome with involvement of cranial nerves have been described. MFS is a rare, acquired nerve disease that is considered to be a variant of Guillain–Barré syndrome. It is determined by abnormal muscle coordination, paralysis of the eye muscles, and absence of the tendon reflexes. Like Guillain–Barré syndrome, symptoms may be preceded by a viral illness.

## Conclusions

This case highlights several neurological and medical complications from COVID-19-provoked GBS, including AMSAN. Our case should be considered as a variant of GBS (the overlap with MFS), which demonstrated an excellent response to immunoglobulin treatment, suggesting the immune-mediated nature of neuropathy. There should be a high suspicion for MFS when the presentation involves ataxia, areflexia, and ophthalmoplegia since MFS is a rare and poorly understood condition. Early treatment with intravenous immunoglobulin can accelerate recovery and improve clinical outcomes. The early recognition of GBS symptoms is critical, given the associated severe motor disabilities that may seriously limit quality of life and requires an accurate evaluation by neurologists.

## Data Availability

All data generated or analyzed during this study are included in this published article

## Abbreviations

GBS: Guillain–Barré syndrome
MFS: Miller Fisher syndrome
COVID-19: Corona virus disease-2019
SARS-CoV-2/: Severe acute respiratory distress syndrome–coronavirus-2
AMSAN: Acute motor sensory axonal neuropathy
CT: Computerized tomography
UI: Urinary incontinence
CMAP: Compound muscle action potentials
ARDS: Acute respiratory distress syndrome
